# Supplementary material for: Altered brain responses to noxious dentoalveolar stimuli in high-impact temporomandibular disorder pain patients
Source: PLoS One. 2022 Oct 14;17(10):e0266349. doi: 10.1371/journal.pone.0266349 (PMC9565712; doi:10.1371/journal.pone.0266349)
Supplement: Attachment — Submitted filename: Review.docx [file pone.0266349.s001.docx]

**Review
”Responses to Noxious Dentoalveolar Stimuli in High-Impact Temporomandibular Disorder Pain Patients”**

The manuscript by Peck et al. aims to determine brain activations in response to noxious dentoalveolar pressure stimuli in chronic TMD patients as well as the CNS effects of pain impact status. I would certainly like to commend the authors for a very thorough study and well-written manuscript. The best available methods have been used and a good and well-defined population was included. The findings in the present manuscript will enable important future specific studies on the mechanisms that are proposed in the findings.

I have only a few major and a few minor comments, see below.

#### MAJOR POINTS

This is a very, very extensive study. I mean that both from a positive and a challeging aspect. In my mind, this is too much to put into a single manuscript. I would urge the authors to consider dividing the manuscript into two (or more?) separate studies in order to improve the possibilities for the readers to understand all your interesting findings.

The manuscript, in its present form, is more of an hypothesis-generating type than an hypothesis-testing type. Nothing wrong with that, especially regarding brain activation by nociceptive stimuli, but I would suggest to change the approach to an hypothesis-generating type of study. And for the manuscript to suggest hypotheses worth specifically investigating in future studies.

There were no significant between-group findings (although interesting and important within-group findings). The authors should discuss this matter more in detail, separately. In the present manuscript a very short sentence is placed in the Conclusion. But what does this mean? Doesn’t chronic TMD pain elicit more brain activation from this stimuli than in controls, i.e. is chronic TMD pain not related to the activation caused by this acute form of stimuli?

The Conclusion is a repetition of the result more that a generalizing conclusion based on the findings. Please rewrite with point no 2 in mind.

The Figure legends must be in much more detail

#### MINOR POINTS

Row 78	Please consider changing ”central sensitisation” to ”maladaptive central pain modulation” or something similar. Central sensitisation is one (of several) specific mechanism whereas maladaptive central pain modulation is a better descriptive term collectively describing all the changes in central pain processing in chronic pain (sensitisation, desensitisation, somatosensory changes, autonomi changes etc).

Row 138	Provide reference to the IRB-approved script

Row 172-	Provide references to all included instruments

Row 197 & 229	Please provide data for the intervals between the sessions

Row 208	If possible, please provide a figure

Row 358-	As I understand, degree of depression and/or anxiety was not controlled for in the MR analyses. This is common in MR analyses since the known effect of depression/anxiety on brain activation. Please motivate what this was not performed or (maybe better?) recalculate. It is not impossible that the possibilities to find significant between-group differences will increase.

Point 2.4.2	Commends for the extensive and top-notch statistics used!

Rows 452-482	Belongs to M&M (if not stated otherwise in the instructions)
